# Draft Genome Sequence of a *Pantoea* sp. Isolated from a Preterm Neonatal Blood Sepsis Patient

**DOI:** 10.1128/genomeA.00904-14

**Published:** 2014-09-11

**Authors:** K. A. Kropp, A. Lucid, J. Carroll, V. Belgrudov, P. Walsh, B. Kelly, K. Templeton, C. Smith, P. Dickinson, A. O’Driscoll, P. Ghazal, R. D. Sleator

**Affiliations:** a Department of Biological Sciences, Cork Institute of Technology, Bishopstown, Cork, Ireland; b NSilico, Cork, Ireland; c Department of Computing, Cork Institute of Technology, Bishopstown, Cork, Ireland; d Division of Pathway Medicine, University of Edinburgh, Edinburgh, United Kingdom; e Microbiological Diagnostic Unit, Royal Infirmary, University of Edinburgh, Edinburgh, United Kingdom

## Abstract

Herein, we report the draft genome sequence of *Pantoea* sp*.* ED-NGS-1003, cultivated from a blood sample taken from a neonatal sepsis patient at the Royal Infirmary, Edinburgh, Scotland, United Kingdom.

## GENOME ANNOUNCEMENT

*Pantoea* spp*.* are Gram-negative, rare, opportunistic pathogens that can infect immune-compromised patients. They cause urinary infections and blood sepsis (1–3) and specifically *Pantoea agglomerans* has been linked to several outbreaks in neonatal units (1, 4, 5). Preterm neonates are a highly susceptible patient group for bacterial infections (6–8) and rapid detection of blood sepsis and identification of the causative agent are critical to enable proper treatment (9–11). The ClouDx-i project aims to extend current knowledge on circulating pathogenic strains linked with neonatal blood sepsis to inform the development of new and improved molecular diagnostic assays. Herein, we present the draft genome of a *Pantoea* sp*.* strain, isolated from a preterm neonate at the Royal Infirmary, Edinburgh in 2013. Positivity for blood sepsis and species identification were confirmed by classical microbiological identification and characterization techniques.

The isolate was grown overnight at 37°C on Luria broth (LB) agar, and genomic DNA was isolated using Qiagen genomic tips (Venlo, Limburg, Netherlands). Genomic DNA fragments were produced ranging in size from 2 to 10 kb using sonication and subsequently used to produce a non-size-selected genome library using the Nextera mate pair kit (Illumina, San Diego, CA). This library was sequenced on an Illumina MiSeq using MiSeq Reagent kit v3. Genomic sequence assembly, analysis and automated reporting was carried out using Simplicity (12). This approach produced 2,596,947 total reads, resulting in an average 114-fold coverage. The average G+C content was 58.80%. For sequence assembly, we used a *de novo* assembly pipeline based on the Spades 3.10 assembly tool with k-mers K21, K33, K55, K77, K99, and K127 nucleotides, resulting in a total of 133 contigs, of which 49 were >1,000 bp representing 99.02% of sequence information. Post assembly processing was performed by Spades and only scaffolds of length greater than 1,000 bp were considered when estimating genome length as 4,822,832 bp. We annotated the genome with Prokka (13) and used the identified 16S rRNA gene to confirm the species as *Pantoea* sp. A scaffold of the genome was produced with Contiguator2 by mapping the contigs back to several *Pantoea* reference genomes. However, BLASTing the scaffold against the NCBI database could not identify a closely related strain. The genome was then screened using Glimmer3 (14) identifying 4,670 open reading frames (ORFs). The predicted ORFs were compared to the Uniprot-Trembl database using BLASTp for strain identification, mapping 3,495 ORFs to the database. To identify potential virulence factors in the genome we compared a local database built from the VFDB (15) and Victors databases with the BLASTp tool, using a 75% amino-acid sequence identity cut-off while only considering alignments longer than 100 amino-acids, identifying 93 hits.

Samples were handled in accordance with local ethical approval by the ethics committees of the NHS Lothian SAHSC Bioresource and NHS R&D office, Project ID 2011/R/NE/01 and the HSS BioResource Request ID 13/ES/0126.

### Nucleotide sequence accession numbers.

This whole-genome shotgun project has been deposited at DDBJ/EMBL/GenBank under accession no. JPQA00000000. The version described in this paper is version JPQA01000000.
